# An electronic biosensor based on semiconducting tetrazine polymer immobilizing matrix coated on rGO for carcinoembryonic antigen

**DOI:** 10.1038/s41598-022-06976-0

**Published:** 2022-02-22

**Authors:** Sowmya Joshi, K. Aswani Raj, M. Rajeswara Rao, Ruma Ghosh

**Affiliations:** 1 Department of Electrical Engineering, Indian Institute of Technology Dharwad, Dharwad, Karnataka 580011 India; 2 Department of Chemistry, Indian Institute of Technology Dharwad, Dharwad, Karnataka 580011 India

**Keywords:** Health care, Cancer

## Abstract

Point-of-care devices are expected to play very critical roles in early diagnosis and better treatment of cancer. Here, we report the end-to-end development of novel and portable biosensors for detecting carcinoembryonic antigen (CEA), a cancer biomarker, almost instantly at room temperature. The device uses reduced graphene oxide (rGO) as the base conducting layer and a novel poly[(1,4-phenylene)-alt-(3,6-(1,2,4,5-tetrazine)/3,6-(1,2,4,5-dihydrotetrazine))] (PhPTz) as an immobilizing matrix for the CEA antibodies. Judiciously introduced nitrogen-rich semiconducting PhPTz brings multiple advantages to the device—(1) efficiently immobilizes anti-CEA via synergistic H-bonding with peptide and *N*-glycal units and (2) transports the charge density variations, originated upon antibody-antigen interactions, to the rGO layer. The CEA was dropped onto the anti-CEA/PhPTz/rGO devices at ambient conditions, to facilitate binding and the change in current flowing through the sensors was measured. A response of 2.75–33.7 μA was observed when the devices were tested for a broad range of concentrations (0.25 pg/mL to 800 ng/mL) of CEA. A portable read-out circuit was assembled using Arduino UNO and a voltage divider circuit, and a simple algorithm was developed for the classification of the CEA concentrations. The prediction accuracy of the interfacing electronics along with the algorithm was found to be 100%.

## Introduction

Rapid and easy diagnostics of diseases have always been crucial for better treatment and prognosis of any disease. It becomes even more critical to detect the disease quickly and at early stages, if it is cancer. The number of cancer cases is increasing annually. In 2018, there were 18.5 million new incidences of cancer which is expected to increase to 29.5 million by the year 2040^1^. The gruesome fact related to this cluster of terminal diseases is that the mortality rates are very high. Cancer is one of the leading causes of death globally^2^. In 2019 trachea, bronchus, and lung cancer claimed 1.8 million lives^3^. The primary reason behind the high mortality rate may be attributed to the diagnosis of cancers at advanced stages^4^. The patients in the early stage of cancer usually observe mild symptoms which overlap significantly with the symptoms of nominal infections, cold, and cough^5^. So, there is resistance among the patients to get themselves tested at an early stage because the current diagnostic methods (computerized tomography scan, bone scan, biopsy, magnetic resonance imaging, positron emission tomography, etc.) are expensive, lab-intensive, and invasive in nature^6^. This prompts for exploring new ways to detect cancer using simple techniques which have the potential to become point-of-care (POC) devices. One of the ways to do so is by developing methods to detect the biomarkers that indicate the presence of the disease at its early stage. Carcinoembryonic antigen (CEA) is one such biomarker that indicates multiple cancers, including lung cancer^7^, oral cancer^8^, colorectal cancer^9^, etc. CEA is also present in healthy human beings but the concentration is much below 5 ng/mL^10^. The concentration of CEA increases in case of any inflammation like cancer cell growth in the body^11^. Hence, developing methods to detect CEA can pave the way for the emerging POC devices for cancer. Different techniques like enzyme-linked immunosorbent assay (ELISA), electrochemiluminescence (ECL), photoelectrochemical-based immunosensors, surface-enhanced Raman scattering (SERS) based sensors have been developed and explored extensively for detecting CEA^12–17^. All these techniques capture the antibody-antigen interaction through chemiluminescence, photochemistry, fluorescence, or change in other optical properties. However, these techniques require bulky instruments, expertise to handle the system and capture the interaction and are expensive^18^. Hence, none of these techniques are potential candidates for POC devices of cancer. Antigens and antibodies are charged species and their interaction leads to change in the overall charge density^19^. Thus, a straightforward way to capture the interaction is by observing the change in the device's current. Since the antibody-antigen interaction is hindered by agglomeration, the device may be developed using a base conducting layer that can facilitate anchoring the antibodies on it and when the antibody-antigen interaction happens, the information related to the change in the total charge faithfully should be transferred to the measuring devices through this transport layer. This mandates the base transport layer to have high mobility and good conductivity. Reduced graphene oxide (rGO) is a derivative of graphene having *sp*^2^ hybridized C-atoms arranged in hexagonal rings. rGO has functional groups attached to its edges and basal planes. It exhibits outstanding properties, including high carrier mobilities which are of interest for developing electronic sensors that capture antibody-antigen interactions^20,21^. The other key material of such an electronic sensor is the antibody immobilizing matrix. Typically, the pendant functional groups present on the material's surface help anchor the antibody via non-covalent/covalent interactions^22^. The covalent linkages between the antibody and the immobilizing matrix proved to provide stable bonding between the matrix and the antibody as well as a defined orientation which will enable stronger antibody-antigen interactions. However, such post-modifications to develop covalent bonds bring several challenges during synthesis and reproduction. Also, functionalization of the biomolecules is necessary to develop a covalent bond between the biomolecule and the matrix. Sometimes, the biomolecules lose bioactivity during the functionalization step. Thus, there is a high demand for immobilizing surfaces that can induce strong interactions without the need of producing covalent bonds. As an alternative, the immobilizing matrix which can develop non-covalent interactions came into the limelight owing to their ability to immobilize the antibodies on their surface via non-covalent interactions (H-bonding, Vander Waal interactions, etc.).

The matrix should have the critical ability to immobilize a sufficiently high density of antibodies without hampering the latter’s recognition activity. Most importantly, it must facilitate regio-selective antibody orientation to allow facile interactions between the antibody and antigen^23^. In search of such functional immobilizing materials, a wide variety of materials ranging from inorganic^24–27^ to natural organic materials^28^ that can interact with the antibody via physical adsorption or chemical bonding has been explored^29^. However, all the aforementioned materials suffer from one or more limitations, including lack of structural tunability, expensive, particle agglomeration, etc. As an alternative, the synthetic organic polymers-based immobilizing matrices came into the limelight as they can offer several advantages over the metal counterparts (low cost, versatile synthetic conditions, stability, structural tunability, and ease of incorporation of the desired functional groups to immobilize the targeted enzyme)^24^. Thus, an extensive array of organic polymers has come into existence. But the majority of the reported polymers are insulators (having an *sp*^3^-hybridized backbone) and thus, their role in the sensor is purely an immobilizing matrix. On the other hand, semiconducting π-conjugated organic polymers as the immobilizing substrates^28^ can anchor the antibodies and also mediate the redox changes that occur during antibody-analyte interactions, to the base conducting layer^30^.

Tetrazine is an aromatic heterocyclic benzene-like compound with an electron-deficient character (LUMO energies ~ 3.15 eV)^31,32^ . Relying on the outstanding optical and electronic properties, several π-conjugated tetrazine oligomers and polymers have been developed and explored for various applications spanning photocatalysis to biological tagging^29^. However, to the best of our knowledge, tetrazine-based compounds have never been utilized as immobilizing matrices for biosensors. *With the electron-deficient functional nitrogen atoms in the core, tetrazine moiety can induce stronger non-covalent (electrostatic/hydrogen bonding) interactions with the antibodies, subsequently can immobilize them effectively and can also play an efficient mediator role.*

This paper reports an end-to-end development of a novel and simple device fabricated using rGO as the base charge transport layer and a novel semiconducting nitrogen-rich tetrazine polymer as an immobilizing matrix for the anti-CEA, owing to the unique ability to develop strong H-bonding interactions with the antibody. The anti-CEA/PhPTz/rGO device demonstrated pronounced biosensing activity towards one of the important biomarkers of cancer. The binding of antigens on the antibodies happened in ambient conditions just by dropping the former onto the latter which makes it different from electrochemical sensors. The current flowing through the biosensor was measured before and after binding the antigen onto the device by applying a DC excitation of 1 V. The change in current was recorded and sensors' responses were found for five different concentrations of CEA at room temperature (25 °C). In an attempt to make the device portable, a simple readout circuit has also been assembled and tested for the accurate determination of the CEA concentration. The results have been presented and discussed in detail in the subsequent sections.

## Materials and methods

### Materials

CEA antigen (ab742) and monoclonal anti-IgG CEA antibodies (ab133633) were purchased from Abcam. Indium tin oxide (ITO) coated sheets (resistivity of ~ 20 Ω/sqm) were purchased from Shilpa Enterprises, India. Aqua regia was prepared in the lab using hydrochloric acid and deionized (DI) water. Graphite powder (282863 Sigma Aldrich) and *l*-ascorbic acid (102312826 Sigma Aldrich), sodium nitrate (So5825 Reachem Laboratory Chemicals Pvt Limited), potassium permanganate (Po4050 Reachem Laboratory Chemicals Pvt Limited). 1,4-dicyanobenzene (D76722 Sigma Aldrich), sulphur powder extra pure 98% (06186 LOBA Chemie), hydrazine hydrate (60%) (4080D LOBA Chemie), sodium nitrite 98% AR/ACS (05954 LOBA Chemie), isoamyl nitrite (0109125 Spectrochem), and hydrogen peroxide (00181 LOBA Chemie) were procured from different suppliers.

### Synthesis of rGO

Graphene oxide (GO) was prepared using the modified Hummer’s method^33^ and was reduced chemically using ascorbic acid to get the rGO. Briefly, GO dispersion (0.1 mg/mL) was taken, ascorbic acid (AA, 4 g) was added to it and was stirred with a magnetic stirrer for 40 min, at 60 °C. The reduced product was centrifuged at 2000 rotations per minute (rpm) for 60 min and the precipitate was collected. Next, the black dispersion was stirred for 40 min, at 60 °C and centrifuged at 2000 rpm to collect the final product. The collected rGO was then washed with ethanol and distilled water several times and dried at 120 °C for 24 h.

### Synthesis of polymer-based immobilizing matrix

#### Synthesis of Poly[(1,4-phenylene)-alt-(3,6-(1,2,4,5-tetrazine)/3,6-(1,2,4,5-dihydrotetrazine))] (PhPTz)

To a 25 ml round bottom flask, in nitrogen ambience, were added 1,4-dicyanobenzene (200 mg, 1.56 mmol), sulphur powder (200 mg, 6.24 mmol), hydrazine hydrate (75%) (0.7 ml, 15.6 mmol), and 10 ml ethanol. The reaction mixture was refluxed with continuous stirring. The initial clear solution gradually turned turbid and subsequently, yielded plenty of solid. After three days, the reddish-brown solid was collected via filtration and used in the next step without purification.

#### Oxidation methods carried out with various oxidizing agents

Subsequently, the oxidation was carried out by reacting the solid with NaNO_2_ (500 mg) in water: acetic acid (2:1) at ice-cold conditions for 24 h. The resultant solid was filtered and washed with hot dimethylformamide (DMF) and subjected to Soxhlet extraction with toluene and ethanol for 48 h and then dried in a vacuum. The reddish-brown solid (PhPTz) with a yield of 76% was isolated.

Note: Oxidation with other oxidizing agents such as nitric oxide (NO), H_2_O_2_, isoamyl nitrite yielded only PhPTz.

#### Synthesis of Poly[(1,4-phenylene)-alt-(3,6-(1,2,4,5-dihydrotetrazine))] (PhUTz)

To a 15 ml pressure vessel were added 1,4-Dicyanobenzene (200 mg, 1.56 mmol), sulphur powder (200 mg, 6.24 mmol), hydrazine hydrate (75%) (0.7 ml, 15.6 mmol), and 10 ml ethanol, and the reaction mixture was heated at 80 °C for three days. The resultant solid was filtered and washed with hot DMF. Soxhlet extraction was carried out using toluene and ethanol for 24 h each which eliminated the oligomeric materials. The solid was dried in a vacuum to achieve 80% yields of PhUTz as an orange solid.

#### Synthesis of model compound (3,6-diphenyl-1,2,4,5-tetrazine) (MTz)

The model compound was synthesized following the steps reported in the literature^34^. ^1^H-NMR (CDCl_3,_ ppm): δ 8.65 (t, 4H), 7.65–7.59 (m, 6H); ^13^C-NMR (CDCl_3_, ppm): δ 164.0, 132.7, 131.8, 129.3, 128.

### Fabrication of biosensor

**Figure 1:**
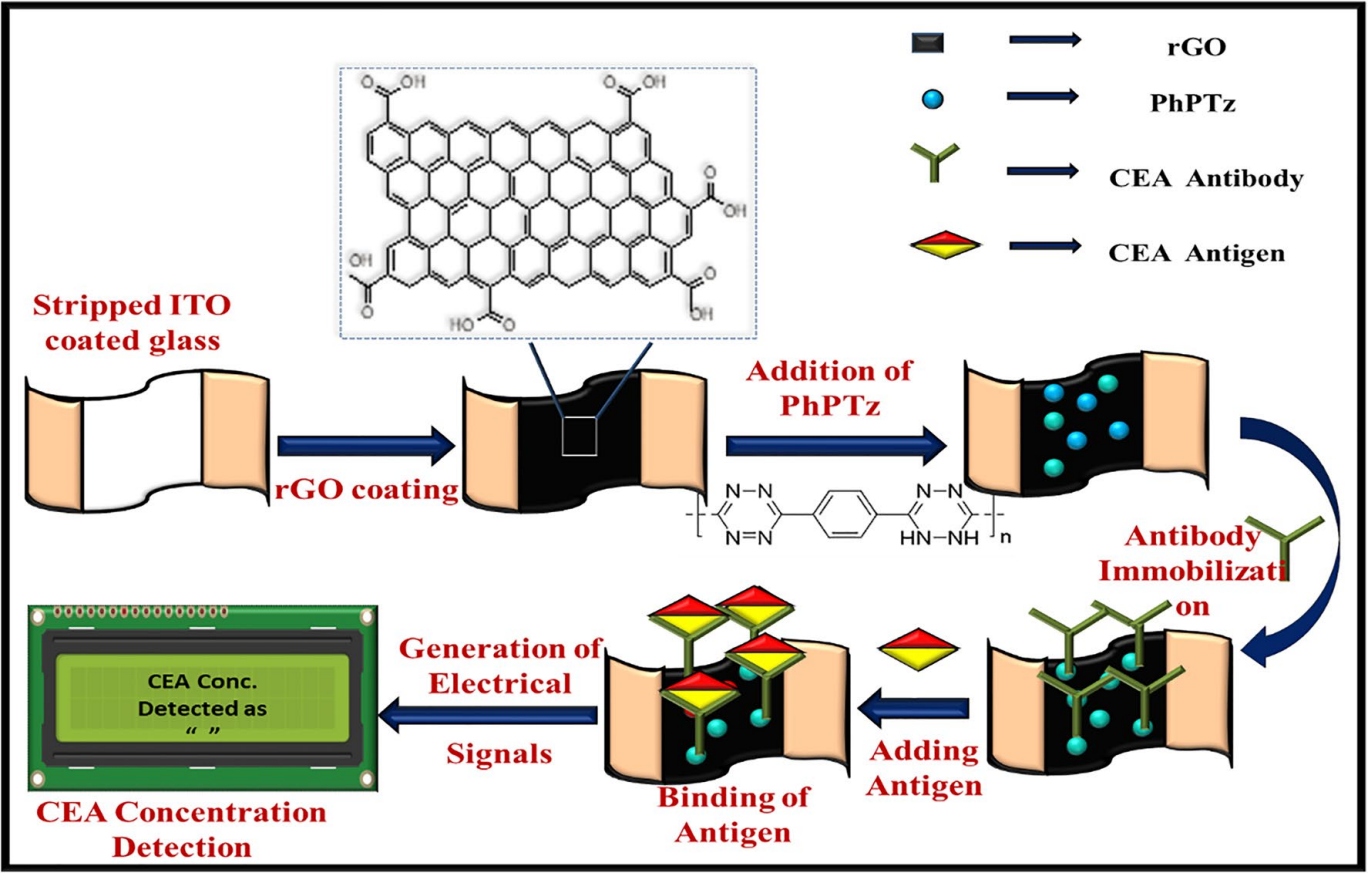
Schematic showing fabrication steps of the electronic biosensor.

ITO coated glasses of dimension 25 mm X 25 mm X 1.1 mm were utilized as substrates for fabricating the biosensors. These substrates were cut into four equal pieces to get one biosensor. Conducting ITO layers were needed at two edges of the device for the biosensor fabrication. Hence, the ITO film of a width of around 1 mm was etched out from the centre of the substrate using aqua regia. These stripped ITO/glasses were then washed with DI water, dried and the resistances across the two ITO electrode strips were checked to ensure proper removal of the ITO film from the centre of the device. Such stripped substrates are open-circuited and hence, no measurable resistances could be observed. The stripped ITO/glasses were coated with rGO (15 μL) and dried at room temperature for 30 min in air. The synthesized polymers (PhPTz and PhUTz) were dispersed in ethanol (10 mg/mL). 2 μL of the dispersion was coated over the rGO layer and then dried in air at 25 °C for 15 min, such that the ethanol added in the polymer gets vaporized. Next, CEA antibodies (6 μL) were drop cast onto the PhPTz/rGO devices and incubated at 4 °C for an hour. After the first incubation, the samples were retrieved and washed with phosphate buffer saline (PBS) solution to remove the unbound antibodies and to eliminate the agglomeration of the antibodies on the surface of the biosensors. These devices were tested for eight different concentrations (0.25 pg/mL to 800 ng/mL) of CEA. CEA (6 μL) of each concentration was dropped onto the anti-CEA/PhPTz/rGO devices followed by the final incubation at 4 °C for an hour. The steps followed for fabricating the electronic sensors are shown schematically in Fig. 1.

### Material characterizations

Park System NX 10 atomic force microscope (AFM) was used to get the thickness of the GO flakes. Carl Zeiss Gemini 300 field emission scanning electron microscope (FESEM) was used to study the surface morphologies of the different layers present in the biosensors. The Oxford energy dispersive spectroscopy (EDS) attached with the FESEM was employed to study the elemental composition of the PhPTz/rGO layers and to ensure the presence of the polymer in the sensor device after several washing steps. Agilent Cary 5000 UV-Vis-NIR spectrophotometer was employed to study the optical properties of the synthesized GO, rGO, polymer, and immobilized antibodies on polymers. The NMR spectra for polymer structural characterizations were performed with a Jeol Resonance ECZ-400R Spectrometer. Infrared spectra were recorded on Nicolet Impact-400 FTIR spectrometer. Solid samples were recorded as KBr wafers and analysed.

### Electrical characterizations

#### Sensor characterizations

For sensing the antigen, 1 V was applied across the two ITO electrodes and the corresponding current flowing through the devices was measured (Fig. 1). The constant voltage was applied using a GPD-3303D regulated power supply (RPS). Keithley data acquisition unit (DAQ6510) was used to measure the electrical current generated at different stages of the device fabrication. The obtained data were continuously logged on to the computer through a graphical interface provided with the DAQ.

#### Read-out circuit for portable sensor

To develop a portable biosensor, it was necessary to replace the benchtop equipment with a miniaturized and compact interfacing read-out circuit. A simple readout circuit was, therefore, assembled by using an Arduino Uno board, a voltage divider circuit to maintain a constant voltage of 1 V across the biosensors, and a liquid crystal display (LCD). Also, an attempt to quantify the antigen concentrations was made by developing an algorithm and loading it onto the Arduino Uno board. The wired setup was validated by testing biosensors with different concentrations of CEA.

## Results and discussion

### Material characterizations

**Figure 2:**
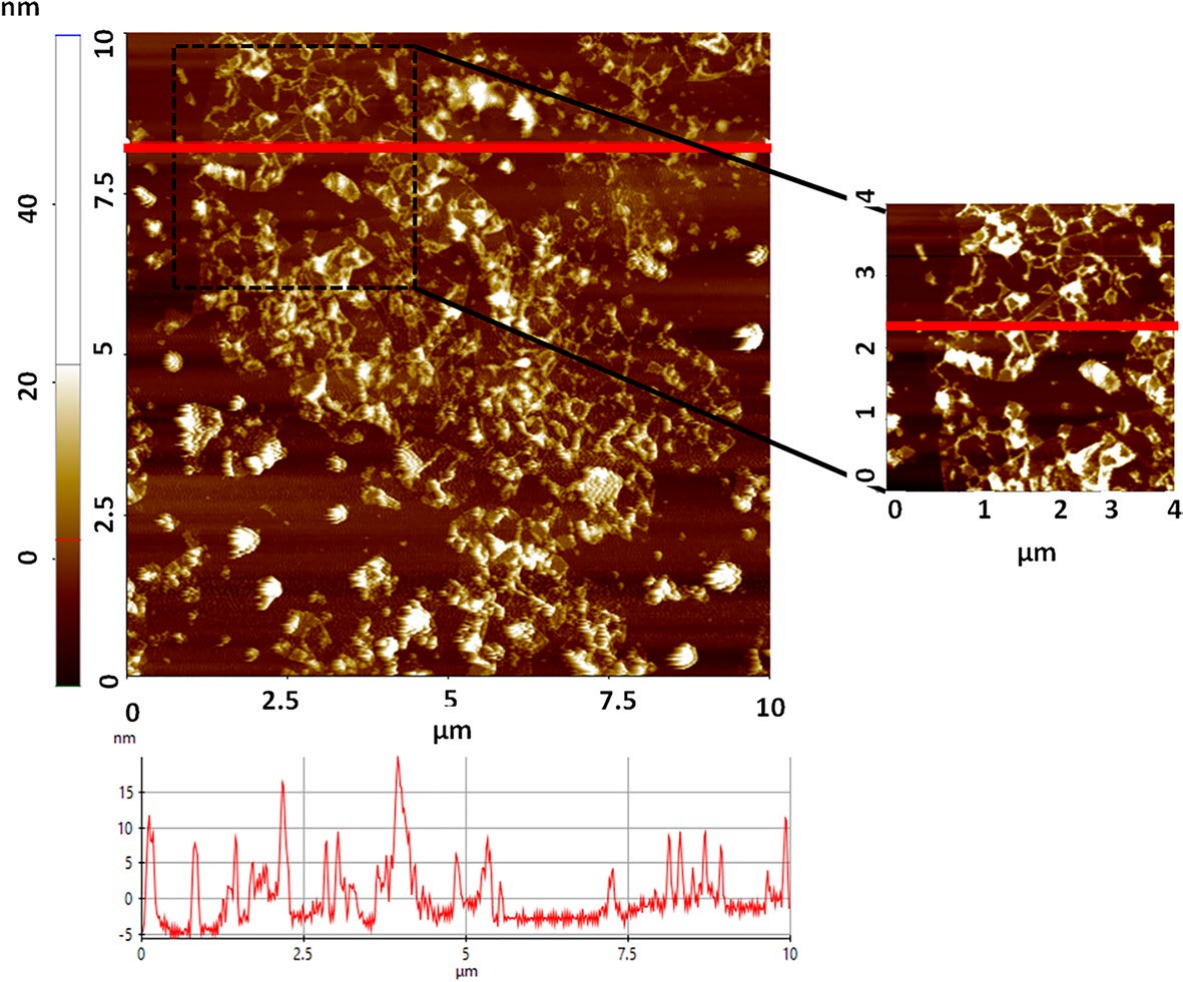
AFM image of GO sheets.

The first step of rGO synthesis was synthesizing GO, which was done using a top-down approach, and hence, it was necessary to ensure the exfoliation. Figure 2 shows the AFM image of the exfoliated GO sheets. The average thickness of the GO sheets was found to be around 5 nm which ensured that the sheets were few-layered^33^.

**Scheme 1:**
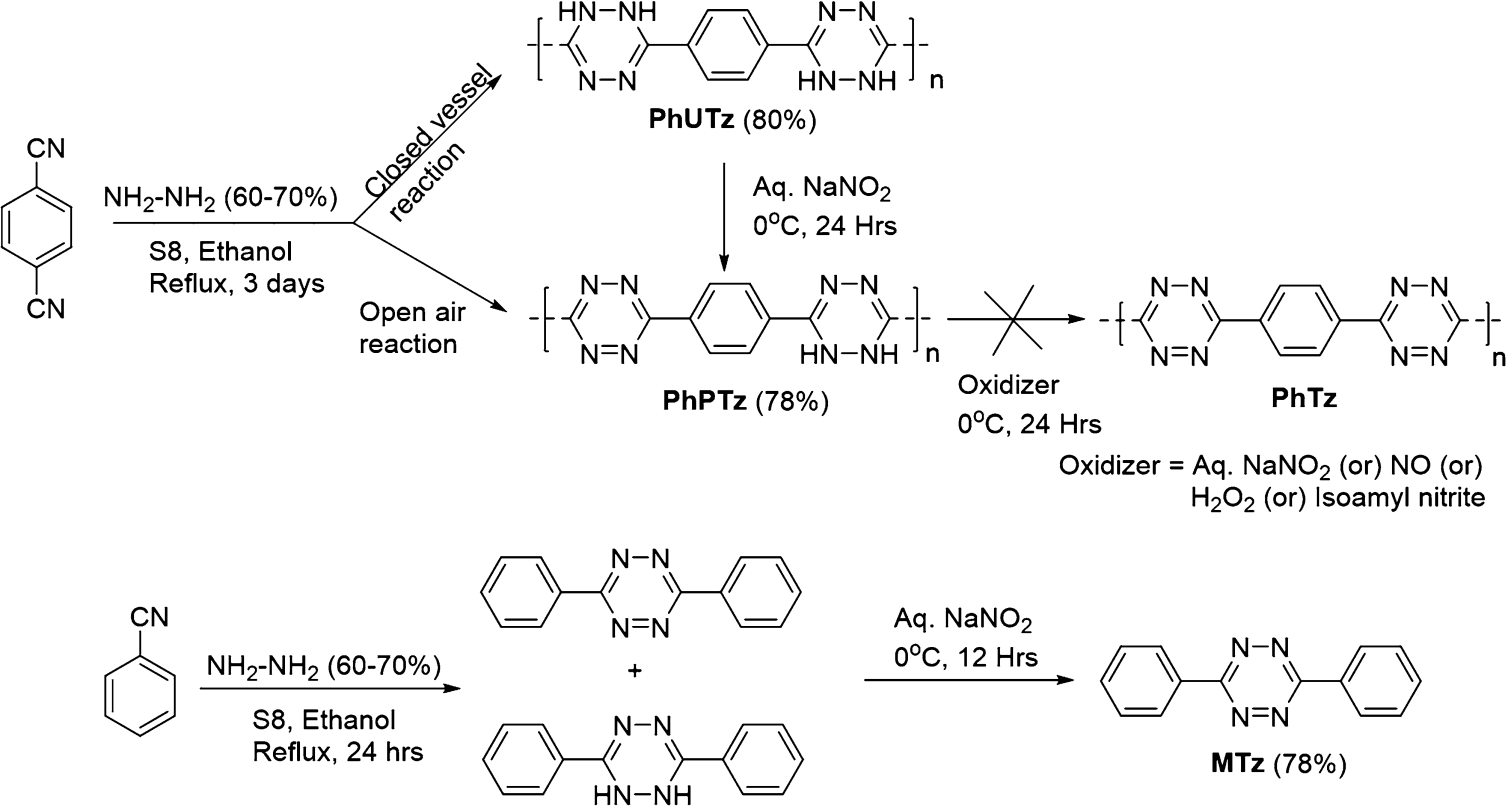
Synthesis of tetrazine polymers (PhPTz and PhUTz) and the model compound (MTz).

On the other hand, the novel tetrazine polymer-based immobilizing matrix was synthesized by following a two-step synthetic process. In the first step, 1,4-dicyanobenzene was polymerized by reacting with hydrazine hydrate (75%) and sulphur in ethanol in an open-air atmosphere at the reflux temperature for three days (Scheme 1). The resulting material was then subjected to oxidation with aq. NaNO_2_ to achieve the fully oxidized tetrazine polymer (PhTz). But interestingly, the reaction yielded a partially oxidized (PhPTz) polymer. Moreover, attempts to further oxidize PhPTz to PhTz with several oxidizing agents such as NO, H_2_O_2_, isoamyl nitrite also met with no success. It has been observed that the material formed during the cyclization in the first step undergoes air-promoted oxidation to yield PhPTz (Fig. S1). It strongly proves that PhPTz is sufficiently high electron-deficient thus, it does not favour further oxidation. To support this hypothesis, we have carried out the density functional theory (DFT) calculations and evaluated the HOMO–LUMO energy levels of three structurally relevant model compounds that possess two dihydrotetrazine (M1), mono-dihydrotetrazine/tetrazine (M2), and two-tetrazine (M3) units. The energy levels of these model compounds highlight that as the number of electron-deficient tetrazines increases, the HOMO energies consistently reduce from -5.66 eV (M1) to -5.92 eV (M2) and -6.28 eV (M3) (Fig. S2) which makes the oxidation harder in the order of M3 > M2 > M1. Thus, PhPTz having several tetrazines in the backbone is expected to possess even lower HOMO energy levels and may resist further oxidation. We also attempted a different synthetic route to obtain PhTz. In this route, initially a fully unoxidized polymer, Poly[(1,4-phenylene)-alt-(3,6-(1,2,4,5-dihydrotetrazine))] (PhUTz) was synthesized via closed-container reaction condition (Scheme 1) which was later subjected to oxidation with aq. NaNO_2_. However, this route also led to the formation of PhPTz only. All the above results suggest that PhPTz is the energetically most stable product and does not lead to further oxidation. Next, we synthesized a solvent-soluble diphenyl tetrazine compound (MTz) using a similar synthetic strategy as a model compound to confirm the formation of the polymer.

The formation of the GO, rGO, and tetrazine polymers was verified by FTIR spectroscopy. It can be observed in Fig. S3a that GO shows numerous peaks of oxygenated groups^35,36^. The vibration peaks of GO appear at 2500–3500, 1724.04 ,1251.5, 1600.6 cm^−1^ and may be attributed to hydroxyl (OH), carbonyl (C = O), alkoxy (C–O–C), and carboxylic (COOH) groups, respectively^37^. The vibration peak at 1724 cm^−1^ disappears in the rGO and a new peak at 2921.6 cm^−1^ appears indicating that the carbonyl groups are reduced to methylene (-CH_2_-). It can be also observed that the intensity of the peak appearing at 1600.627 cm^−1^ decreases drastically in the rGO spectra suggesting that most of the carbonyl groups are reduced^38^. The oxygen functional groups are expected to aid in the immobilization of antibodies but at the same time, higher conductivity is a pivotal characteristic required for the base transport layer. The conductivity of the rGO sample increased significantly only after reduction as is evident from the I-V characteristics of GO and rGO (Fig. S3b).

**Figure 3:**
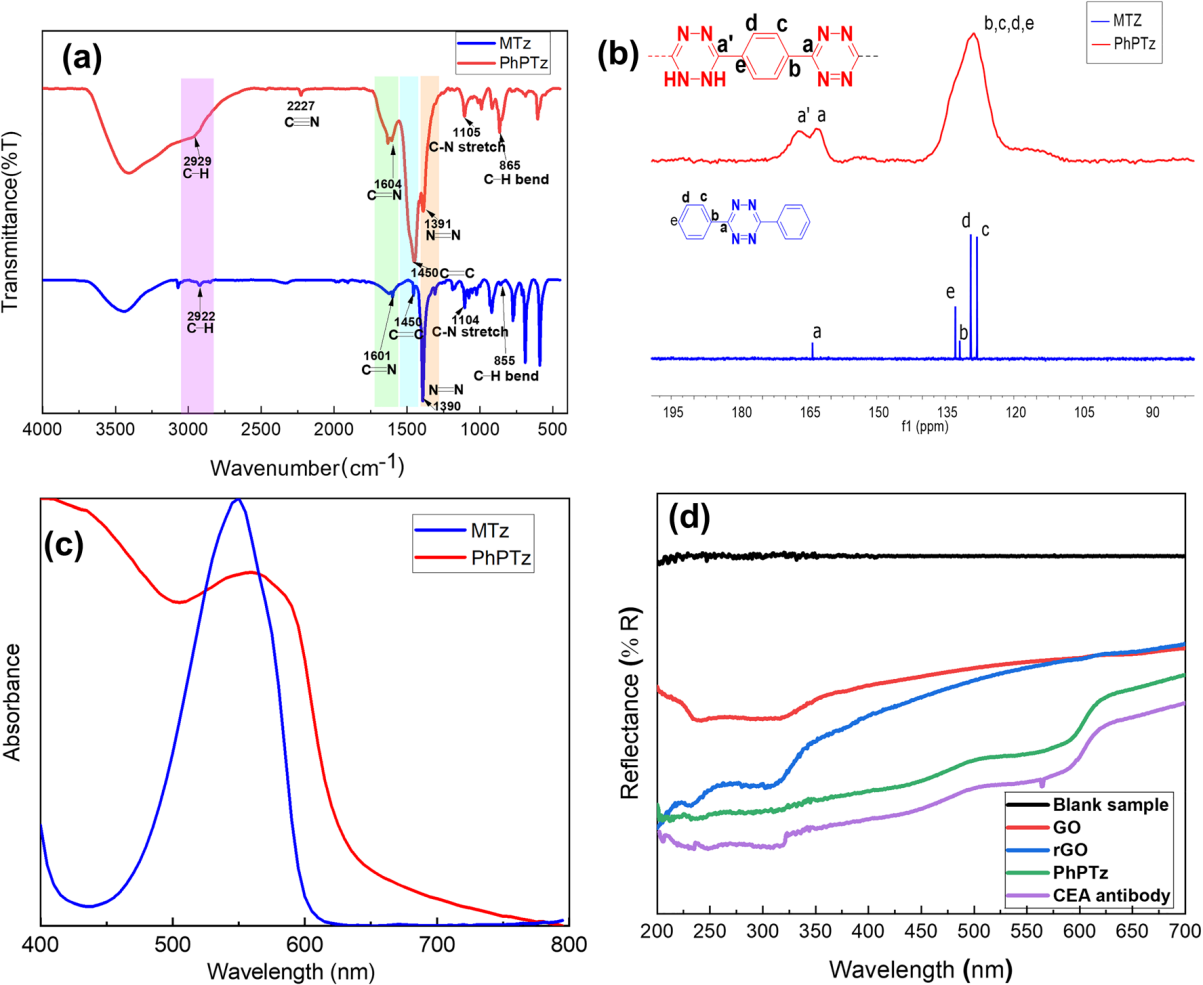
IR and NMR characterization of polymer: comparison of (**a**) IR spectra, (**b**) ^13^C NMR spectra of tetrazine polymer (PhPTz) and the model compound (MTz); (**c**) Comparison of the diffuse reflectance spectrum of PhPTz and the solution state absorption spectrum of MTz; (**d**) UV–Visible reflectance spectra of blank sample (only glass), GO, rGO, PhPTz, and anti-CEA/PhPTz/rGO thin films.

The FTIR spectrum of PhPTz in Fig. 3a shows the characteristic C = N and N–N stretching^39^ at 1604 cm^−1^ and 1391 cm^−1^, respectively, confirming the presence of tetrazines. The C = C and C-H stretching belonging to the phenylene unit appeared at 1450 cm^−1^ and 2920 cm^−1^, respectively. Additionally, the peak at ~ 2200 cm^−1^ corresponds to the residual nitrile (-C≡N) end group. Very weak signal intensity corroborates with a low concentration of -C≡N groups and a longer polymeric chain. The C-N stretching and C-H bending of the tetrazine and the phenylene units of the polymers have been observed at ~ 1105 cm^−1^ and ~ 860 cm^−1^ respectively. All the IR signals of the polymer are fully consistent with those of the model compound (MTz). The peaks exhibited by PhUTz are also in line with those of PhPTz (Fig. S3b), confirming the existence of C = N and N–N stretching in PhUTz. The chemical structures of the polymers have been further confirmed by solid-state ^13^C-NMR spectroscopy. PhPTz showed a characteristic signal at ~ 162 ppm and ~ 166 ppm corresponding to the tetrazine carbons (C = N) and dihydro-tetrazine carbons (C-NH), respectively (Fig. 3b). On the other hand, PhUTz exhibited only one strong signal at ~ 166 ppm, indicating that the polymer is in the dihydrotetrazine form (Fig. S4). Both the polymers' phenylene carbon signals appeared between 120–140 ppm. Thermal gravimetric analysis (TGA) of PhPTz showed an onset of thermal decomposition temperature at around 260 °C. With an increase in the temperature, the polymer showed a continuous mass percentage loss and reached 20 wt% at 500 °C (Fig. S5). It has been proved that rGO can immobilize the anti-CEA via the functional groups such as –COOH, -C = O, however, the concentration/density of such groups is low. As a result, the density of anti-CEA immobilizing on the rGO surface are also low, leading to poor sensing. To overcome this limitation, we have introduced tetrazine-based organic polymer having a high concentration/density of N-heteroatoms which is expected to anchor the anti-CEA on the sensor surface, more effectively.

PhPTz is an intensely coloured powder that can absorb most of the visible light energy. Figure 3c shows the diffused reflectance spectrum of the polymer, which reveals a broad absorption band ranging from 400–650 nm (λ_max_ = 575 nm), which is relatively red-shifted compared to that of MTz (λ_max_ = 550 nm). Thus, rGO behaves like a semiconductor material after reduction. For PhPTz polymer, the optical bandgap was observed to be 1.90 eV (Fig. S6). The low bandgap suggests that the polymer possesses semiconducting properties. The optical bandgap of GO and rGO to estimated be 2.46 eV and 1.56 eV respectively (Figs. S7, S8) from the Tauc plot. The decrease in the band gap of the rGO is mainly due to the reduction of oxygen groups^40,41^. The sensing layers were characterized at different stages of the device fabrication using UV–Vis spectroscopy. As depicted in Fig. 3d the reflectance profile of anti-CEA/PhPTz/rGO shows all the peaks exhibited by rGO and tetrazine, confirming the presence of all the materials in the sensor device.

**Figure 4:**
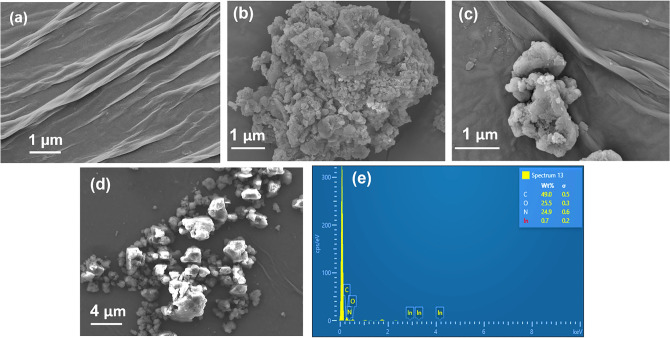
FESEM image of (**a**) rGO, (**b**) PhPTz, (**c**) PhPTz/rGO, (**d**) anti-CEA/PhPTz/rGO, (**e**) EDS spectra of PhPTz/rGO.

Next, as sensing is a surface mechanism, it was imperative to study the surface morphologies of the different constituents of the biosensor. Figure 4 presents the FESEM image of rGO, PhPTz, PhPTz/rGO, and anti-CEA/PhPTz/rGO. rGO is a two-dimensional material that when coated on any substrate exhibits a crumpled and wrinkled sheet-like structure (Fig. 4a). PhPTz displays large flakes packing together to form big lumps. This can be attributed to the rigid, planar, and linear structural characteristics of PhPTz which may facilitate strong π-π interactions and lateral H-bonding interactions with the neighboring polymeric chains, leading to such morphology. The wrinkled rGO sheets and the agglomerated polymer coexist in the PhPTz/rGO sample (Fig. 4c). The agglomeration of the polymer can be attributed to the high surface energy of molecules. The cross-linkage of the polymer is still visible in the composite film. CEA antibodies are not conducting in nature. Figure 4d shows features with anomalous contrast which is because of the charging effect happening in the anti-CEA/PhPTz/rGO film. The charging effect is observed in the FESEM images when the high energetic electronic beam interacts with a non-conducting surface (CEA antibodies in this case). The tetrazine polymer displayed almost the same morphology in the as-synthesized, PhPTz/rGO and anti-CEA/PhPTz/rGO samples, indicating that the bonding properties of the polymers are retained. EDS spectrum of the anti-CEA/PhPTz/rGO film was acquired to confirm the purity and the presence of all three layers in the sample. Figure 4e shows peaks of C, N, O, and In atoms. The C-atoms are contributed collectively by all three constituents. N-atoms are contributed by the polymer and the CEA antibodies, O-atoms are available with rGO, anti-CEA layers, and the base ITO/glass substrate. In-atoms are contributed by the substrate.

### Biosensor characterizations

Literature reports that in the case of oral squamous cell carcinoma, the concentrations of the CEA in the patients’ saliva were found to be ranging from 20 ng/mL to hundreds of ng/mL whereas this concentration was found to be varying as 22.6 ± 22.1 ng/mL in case of healthy persons^42^. Similar is the case for lung cancer^10^. 20 ng/mL of the CEA can be found in both healthy persons as well cancer patients. Hence, the developed biosensors were tested for different concentrations of CEA varying from 0.25 pg/mL to 800 ng/mL covering the entire range of concentrations found in healthy persons to those of cancer patients. As the current through the electronic sensors decreased in presence of CEA and to nullify the effect of baseline variations of the devices, the response of the sensors was measured as follows:

1 $$Response = I_{o} - I_{target}$$

where I_target_ is the current measured across the sensor in presence of the antigen and I_o_ is the baseline current measured with anti-CEA/PhPTz/rGO polymer.

**Figure 5:**
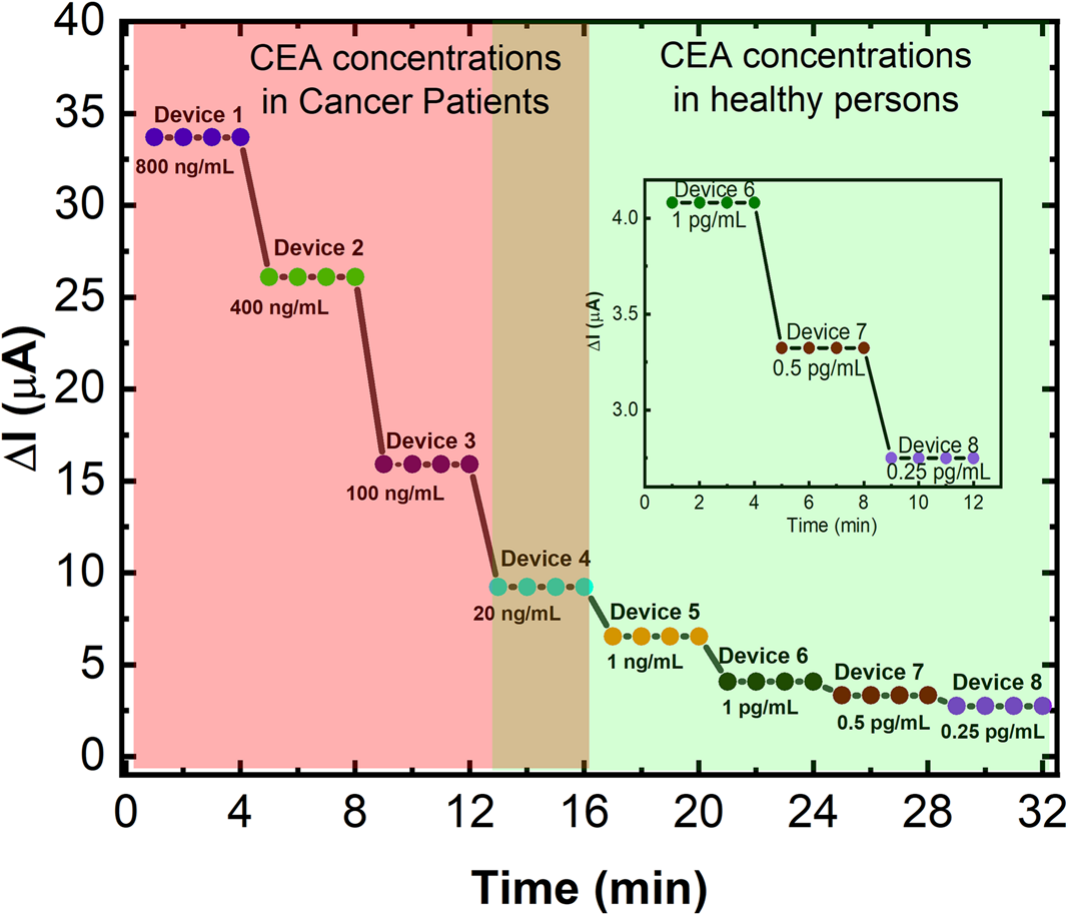
Response of anti-CEA/PhPTz/rGO sensors to 0.25 pg/mL to 800 ng/mL of CEA. Inset shows the response of the anti-CEA/PhPTz/rGO sensors to 0.25 pg/mL to 1 pg/mL of CEA.

The response of the anti-CEA/PhPTz/rGO sensors was found to be excellent and varied from 33.7 µA for 800 ng/mL to 2.75 µA for 0.25 pg/mL (Fig. 5). The responses of the sensors were observed to gradually decrease with a decrease in the concentration of the CEA antigen which indicates the dependence of CEA concentration on the sensors’ responses.

Limit of detection (LOD) of biosensors is a pivotal parameter and is calculated statistically using the relation shown in Eq. (2)

2 $$LOD = \frac{{3 \times rms_{noise} }}{slope}$$

where rms_noise_ is the root mean square of the noise. This is captured through the blank measurements of the sensors (Fig. S9a). slope is found from the linear fitted concentration Vs response plot (Fig. S9b). The details of the LOD calculation have been reported earlier^43^. The rms_noise_ was calculated to be 1.0924 × 10^−7^. The slope was found to be 0.3753 and hence, the LOD of the sensors was calculated to be 8.78 × 10^−7^ pg/ mL.

It was observed during the preliminary studies that the volume of the polymer in the device was affecting the device’s performance. Hence, the optimum volume of the polymer in the biosensor was found by fabricating multiple devices with varied volumes (1–10 μL) of the polymer dispersion and then testing all the devices for 1 pg/ml concentration of CEA. It was observed that 2 µL volume of PhPTz was found to exhibit the best response (4.11 μA) as compared to the rest of the volumes of tetrazine tested (Fig. S10). This might be because the lower volume of the polymer (1 μL) fails to provide sufficient binding sites to the antibody and when the volume of PhPTz is increased beyond 2 μL, it causes agglomeration of the antibodies on the sensor device, which is detrimental to the sensor performance. After optimizing the volume of PhPTz to be used in the sensor devices, all the experiments were conducted with 2 µL of PhPTz.

**Figure 6:**
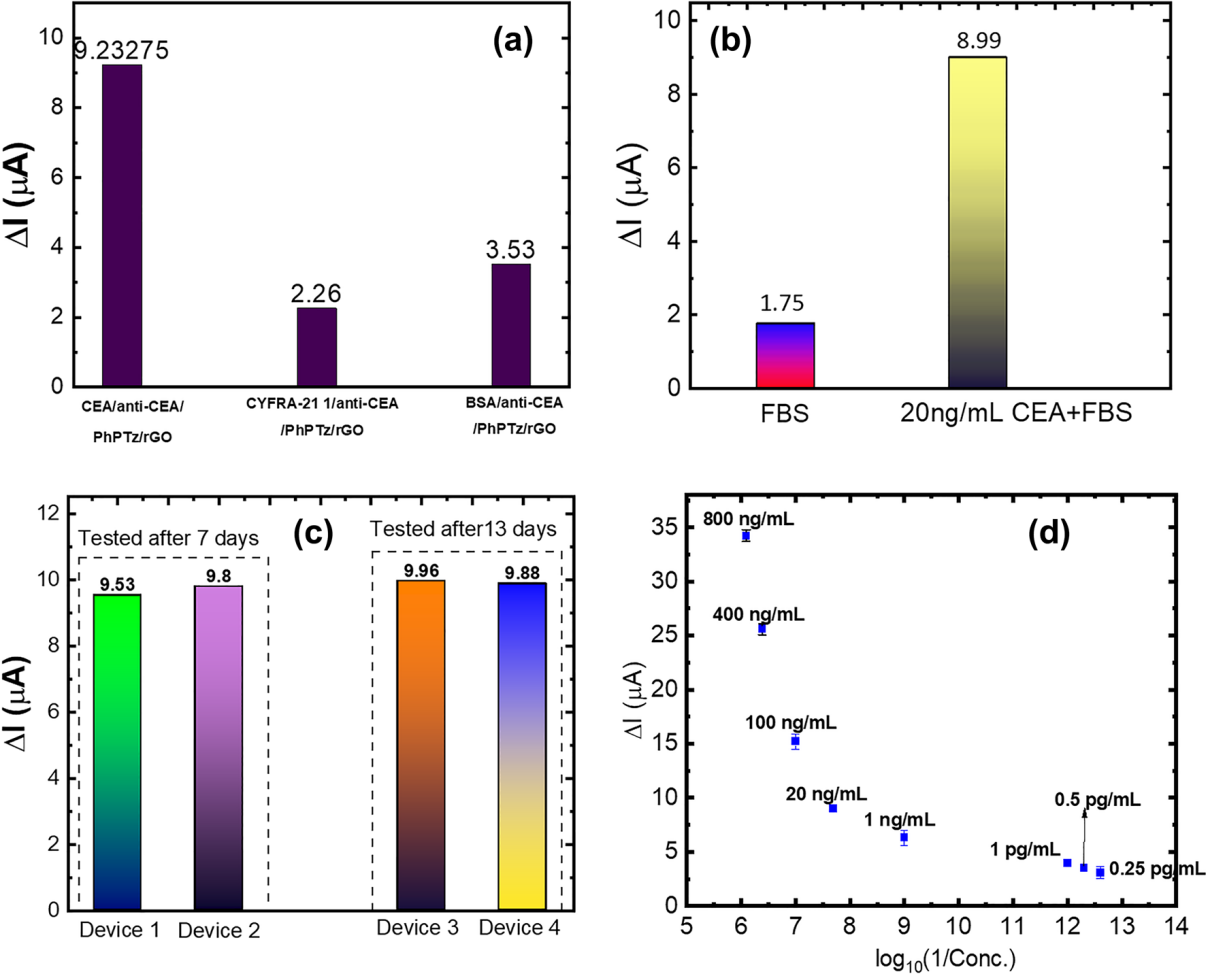
(**a**) Cross-reactivity results for biosensors for 20 ng/mL CEA. (**b**) Response of anti-CEA/PhPTz/rGO to FBS and FBS + 20 ng/mL CEA. (**c**) Stability test results of anti-CEA/PhPTz/rGO sensors to 20 ng/mL of CEA. (**d**) Variation in sensors’ response with different devices having different baseline currents.

In addition to the sensitivity, selectivity is another important parameter of a biosensor. The sensor was tested with CYFRA 21–1 (which is another biomarker of cancer) and bovine serum albumin (BSA) which is present in the human body in abundance, to evaluate the selectivity of the developed sensor. The response of the sensor was found to be negligible as compared to the response demonstrated for 20 ng/mL CEA (Fig. 6a). The selectivity of the biosensors was further confirmed by testing them with fetal bovine serum (FBS) which is very complex in nature with multiple nutritional and molecular agents, essential for cell growth present in it. FBS also resembles the human serum samples which are equally complex. The response of the anti-CEA/PhPTz/rGO sensor to 6 μL FBS was found to be 1.75 µA which indicates the presence of CEA with a concentration lower than 0.25 pg/mL (Fig. 6b). To further ensure the interference immunity of the biosensor, the FBS (6 μL) was spiked with 20 ng/mL CEA (6 μL). The response of the CEA antigen /anti-CEA/PhPTz/rGO sensor was found to be 8.99 µA which is close to the response exhibited by the sensors to the pure CEA antigen (Figs. 5, 6b).

In order to validate that the sensor signals were generated due to antibody-antigen interaction, the biosensors were fabricated coating only rGO and PhPTz layers. These devices were tested by binding the 20 ng/mL and 1 ng/mL of CEA. It can be observed from Fig. S12 that the change in the current was very less when compared to the response of anti-CEA/PhPTz/rGO for 20 ng/mL and 1 ng/mL CEA concentrations. This showed that no significant change in the current through the sensing device was observed unless the antigen–antibody interaction happens.

The biosensors were also tested for their stability by preparing the devices, storing them at 4 °C, and testing them for 20 ng/mL CEA after 7 and 13 days. The responses of the sensors were found to be varying between 9.53 to 9.96 μA (Fig. 6c) which is very close to the response of freshly prepared biosensors (9.25 μA, as shown in Fig. 5).

This paper reports development of an electronic biosensor that could sense different concentrations of CEA just by dropping 6 μL antigen onto the device. The performance of the developed sensors was found to be excellent and was compared with some of the recently reported literature on the detection of CEA using other techniques as listed in Table 1.

**Table 1:** Comparison between different biosensors reported for CEA detection and the ones developed in this research.

Sl. no.	Type of immunosensor	Sensing material	Lowest measured concentration	References
1.	ELISA	–	ng/mL	^35^
2.	Electrochemical immunosensor	Nanopore-based strategy	0.6 ng/mL	^26^
3.	Optical biosensor	Antibody-quantum dot conjugates	0.1 ng/mL	^13^
4.	Colorimetric sensor	Au nanoparticle-decorated Bi_2_Se_3_ nanosheets	160 pg/mL	^16^
5.	Electrochemical immunosensor	Iron oxide nanoparticles decorated PEDOT:PSS	4 ng/mL	^15^
6.	This work	CEA/anti-CEA/PhPTz/rGO	0.25 pg/mL	–

### Sensing mechanism

The novel device developed in this research has multiple layers in it, each of which plays a unique and pivotal role as detailed below:

#### Role of PhPTz

**Figure 7:**
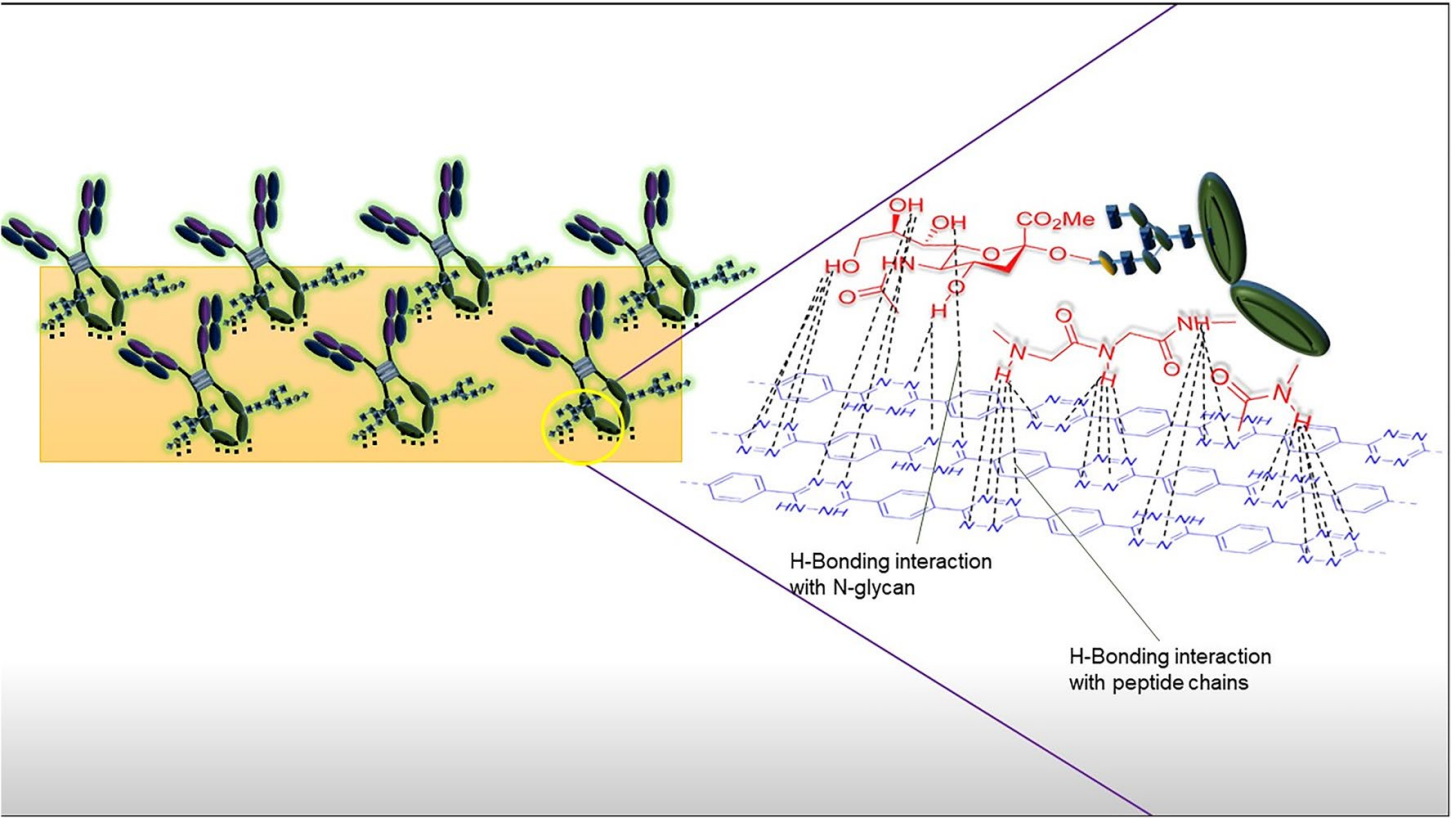
The Schematic diagram depicting the molecular interactions between anti-CEA and the tetrazine polymers.

The high response of the device can be attributed to the electron-deficient tetrazine units loaded polymer (PhPTz) which possesses a novel structural feature to effectively immobilize the anti-CEA. The nitrogen atoms in the tetrazine ring are expected to induce strong H-bonding interactions with the peptide units and the N-glycans at the Fc domain of the anti-CEA (Fig. 7). The synergistic effects are expected to provide stronger bonding interactions between the antibody and the matrix and render a regio-specific conformation to the antibody, leading to an outstanding sensing performance^44^. To prove the importance of the tetrazine units in immobilizing the anti-CEA, we have fabricated a control device with a fully unoxidized polymer (PhUTz) that does not possess tetrazine rings. The response of the anti-CEA/PhUTz/rGO was found to be only 4.32 μA upon exposing the sensors to 20 ng/mL of CEA (Fig. S11) which is less than half the performance of PhPTz. This indeed highlights the role of tetrazine units in effectively immobilizing the anti-CEA.

#### Role of rGO

The antigen–antibody interaction happens because of different forces acting between the epitope of the antigens and the paratope of the antibodies^45^. But the electrostatic force is the one of interest in this case. The antibodies and antigens are oppositely charged species and due to their interaction, clustering happens and the net charge of the clustered species reduces. This change in the net charge on the antibodies is transferred to the rGO layer by the semiconducting polymer. This, in turn, results in the net charge present in the *p-*type rGO layer. Since the device is excited with a DC voltage and owing to the outstanding electronic properties of the rGO layer, the change in the net charge in the biosensor gets reflected as the decrease in current flowing through the anti-CEA/PhPTz/rGO device. The role of rGO in the device is equally critical as that of PhPTz and without the rGO layer in the device, the baseline of the sensor (PhPTz/anti-CEA) was found to be very low (~ nA) and no significant change in current could be observed after incubating the CEA.

### Readout circuit development for portable biosensor

#### Development of the algorithm for accurate quantification of CEA concentration

**Figure 8:**
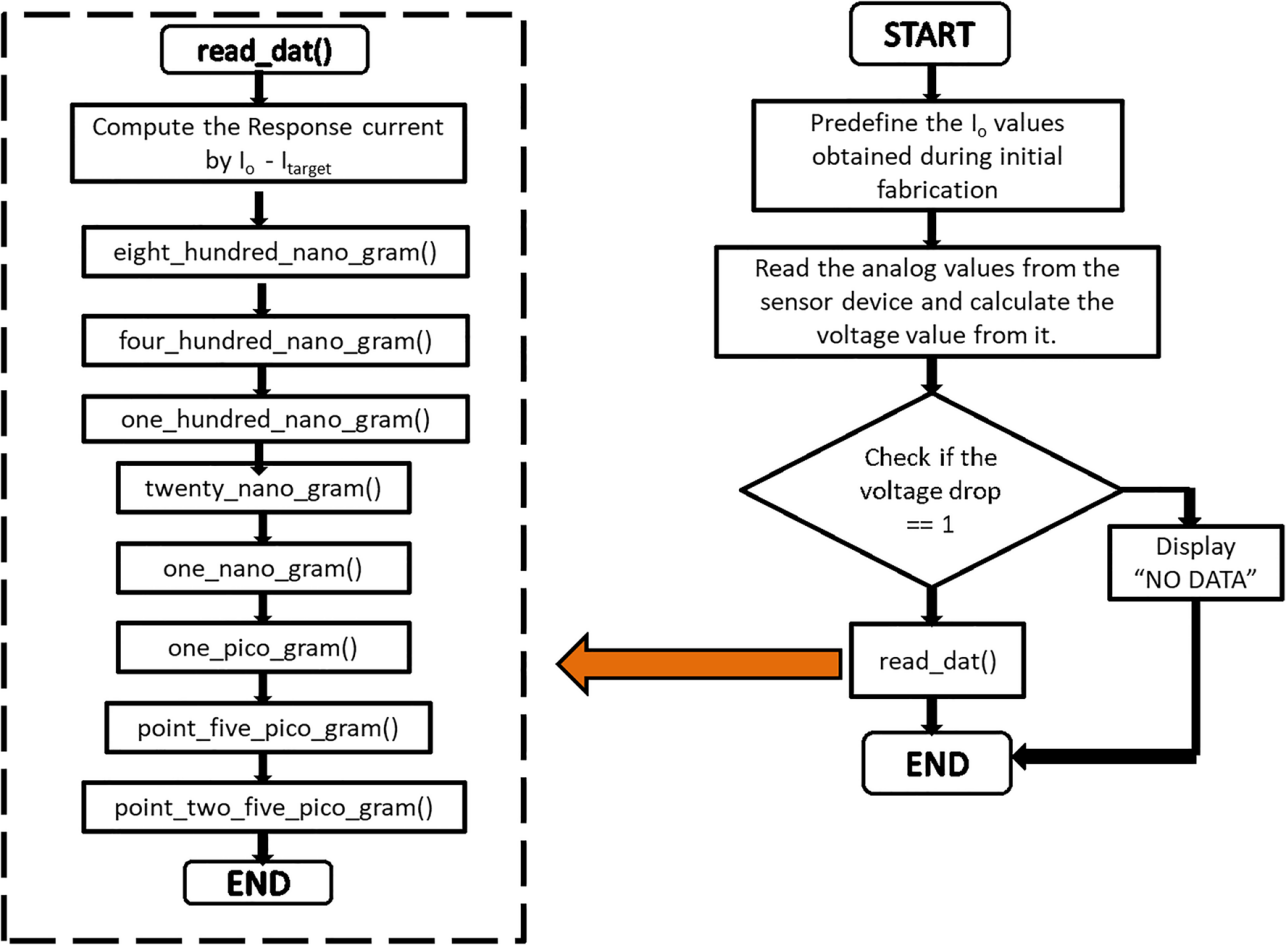
Flowchart of the algorithm developed for the prediction of CEA concentrations.

**Figure 9:**
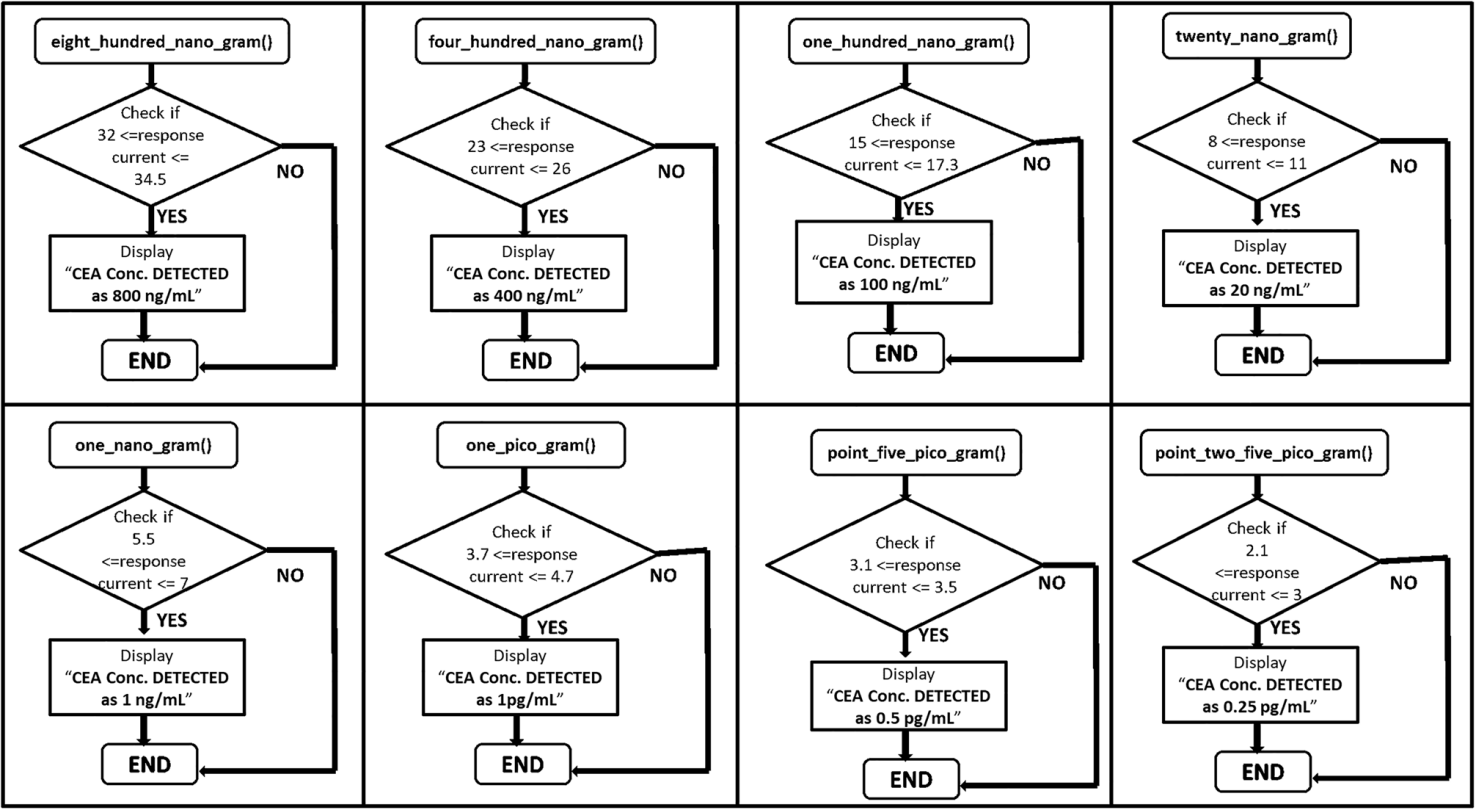
Flowchart of the subroutines used for the prediction of CEA concentrations.

To predict the concentration of the antigen, it was necessary to test multiple devices with varied baseline currents for all the concentrations of CEA and assess the variation in the response of the anti-CEA/PhPTz/rGO devices. Figure 6d shows the variations in the response of the sensors for different concentrations of CEA. A total of 30 devices (3–4 devices for each concentration of CEA) were prepared and tested for the target antigen. As the rGO is chemically reduced, the I_o_ value may change due to many factors, including the number of layers getting coated, but the responses of the sensors were found to be independent of the baseline current. The maximum variation in the response of the biosensors was found to be around 0.7 μA which was observed for 1 ng/mL CEA. Also, the responses of the sensors were found to be non-overlapping across the concentrations of CEA (Fig. 6d). This aided in developing a simple algorithm for the accurate prediction of the CEA concentrations. The logic of the developed algorithm is demonstrated in Figs. 8, 9. The algorithm starts with I_o_ values that should be acquired before incubating the antigen onto the device. As soon as we plug the CEA-bound device with the read-out circuit, the I_target_ values are acquired. The algorithm then calculates the response, I_response_ as I_o_ – I_target_. Post to the calculation of I_response_ value, the program enters into one of the eight subroutines shown in Fig. 9, depending on the conditional check being TRUE. The blocks in Fig. 9 represent the series of condition checks for each subroutine that was created to determine the CEA antigen concentration present in the sensor device. It starts with checking the response values and then finds the correct range of response to which it belongs.

#### Assembling the portable readout circuit

**Figure 10:**
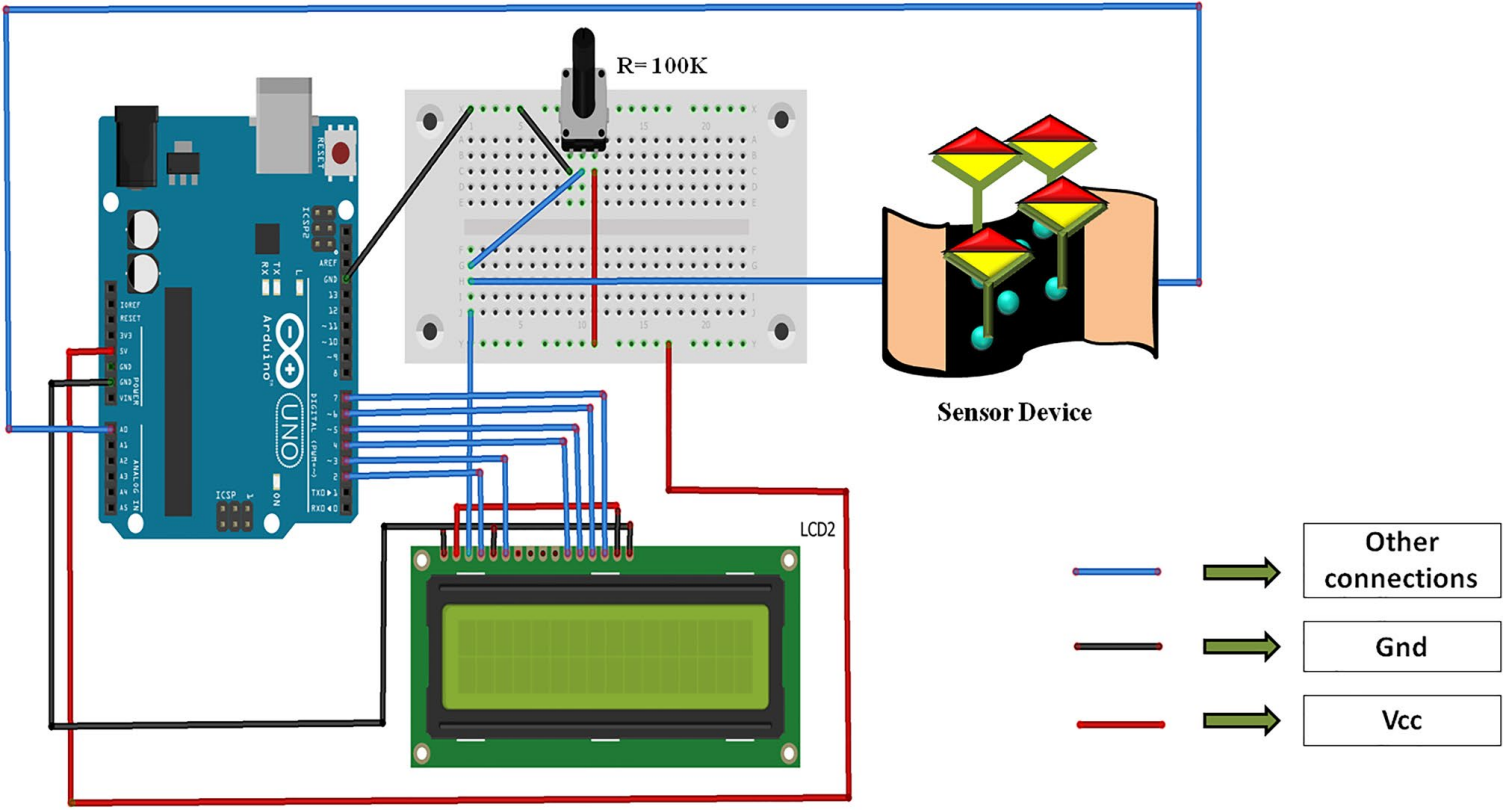
Diagram showing the assembled read-out circuit.

A simple read-out circuit for the developed electronic biosensor was assembled using an Arduino UNO board, LCD, and a potentiometer (Fig. 10). There were a few challenges faced while assembling the circuit. Firstly, Arduino UNO cannot be used directly to measure the current flowing through a device. One of the ways it can be facilitated is by inducing a voltage drop across the device (V), measuring the resistance (R), and finding the current, I as V/R. Secondly, UNO sources 5 V but our sensors were characterized by applying 1 V across them (minimized power consumption). So, a voltage divider circuit was deployed that ensured a drop of 1 V across the biosensor. The next challenge in the circuit assembly was the device-to-device variation in biosensor resistances. The baseline resistance of the biosensor varied with devices. Though this variation didn’t affect the response of the sensors (Fig. 6d), it certainly disturbed the potential divider circuit. Hence, a potentiometer was used to match the resistance value to that of the plugged-in sensor for calculating the I_target_ value accurately. The current, I_response_ hence, captured through the readout circuit was recorded and processed by the algorithm that was loaded onto the microcontroller of the UNO board, to predict the concentration of CEA. This portable circuit reduced the dimensions of the read-out circuit significantly and the circuit was used to read the run time signals generated by seven different sensors and the accuracy of the developed algorithm was found to be excellent with 100%.

## Conclusions

A label-free, rapid, simple, and easy-to-use biosensor was developed for detecting different concentrations of CEA. The sensors employed a 2D rGO layer as the base transport layer, semiconducting PhPTz as the immobilizing agent for the antibodies on rGO, and CEA antibodies. The developed sensors were tested for eight different concentrations (0.25 pg/mL to 800 ng/mL) of CEA at room temperature. The change in the current was recorded as the response of the sensor. The biosensors exhibited a response ranging from 2.75 μA to 33.7 μA for 0.25 pg/mL to 800 ng/mL of the CEA. The responses of the sensors were found to be highly reproducible even when tested on devices having varied baseline currents. A portable read-out circuit was assembled as a step to develop the prototype of the biosensors for the accurate prediction of CEA concentrations. The prediction was facilitated by an algorithm that was developed based on the reproducibility results of the sensors. It is believed that this work will lead to the development of a commercial sensor that will be capable of detecting CEA and thus, indicating the occurrence of terminal diseases at their early stages.

## Supplementary Information

Supplementary Figures.

## References

[1] National Cancer Institute. Statistics at a glance: The burden of cancer in the United States. *Cancer Stat.* OMB No.: 0925-0642 (2017).

[2] Rana JS, Khan SS, Lloyd-Jones DM, Sidney S (2020). Changes in mortality in top 10 causes of death from 2011 to 2018. J. Gen. Intern. Med..

[3] WHO. The top 10 causes of death—Factsheet. *WHO Reports* 1–9 (2020).

[4] Siegel MPH RL, Miller MPH KD, Fuchs BS HE, Jemal DVM A (2021). Cancer Statistics 2021. CA Cancer J. Clin..

[5] Jemal A (2017). Annual Report to the nation on the status of cancer, 1975–2014, featuring survival. J. Natl. Cancer Inst..

[6] Prabhakar B, Shende P, Augustine S (2018). Current trends and emerging diagnostic techniques for lung cancer. Biomed. Pharmacother..

[7] Arya SK, Bhansali S (2011). Lung cancer and its early detection using biomarker-based biosensors. Chem. Rev..

[8] Zheng J (2018). Clinical value of Naa10p and CEA levels in saliva and serum for diagnosis of oral squamous cell carcinoma. J. Oral Pathol. Med..

[9] Björkman K (2021). A prognostic model for colorectal cancer based on CEA and a 48-multiplex serum biomarker panel. Sci. Rep..

[10] Vincent RG, Chu TM, Fergen TB, Ostrander M (1975). Carcinoembryonic antigen in 228 patients with carcinoma of the lung. Cancer.

[11] Sørensen CG, Karlsson WK, Pommergaard HC, Burcharth J, Rosenberg J (2016). The diagnostic accuracy of carcinoembryonic antigen to detect colorectal cancer recurrence—A systematic review. Int. J. Surg..

[12] Qi H, Zhang C (2020). Electrogenerated chemiluminescence biosensing. Anal. Chem..

[13] Wang H, Wang X, Wang J, Fu W, Yao C (2016). A SPR biosensor based on signal amplification using antibody-QD conjugates for quantitative determination of multiple tumor markers. Sci. Rep..

[14] Wang H (2016). Photoelectrochemical immunosensor for detection of carcinoembryonic antigen based on 2D TiO_2_ nanosheets and carboxylated graphitic carbon nitride. Sci. Rep..

[15] Kumar S (2019). Electrochemical paper based cancer biosensor using iron oxide nanoparticles decorated PEDOT:PSS. Anal. Chim. Acta.

[16] Xiao L (2017). Colorimetric biosensor for detection of cancer biomarker by Au nanoparticle-decorated Bi_2_Se_3_ nanosheets. ACS Appl. Mater. Interfaces.

[17] Kazemi SHK, Ghodsi E, Abdollahi S, Nadri S (2016). Porous graphene oxide nanostructure as an excellent scaffold for label-free electrochemical biosensor: Detection of cardiac troponin I. Mater. Sci. Eng. C.

[18] Sandbhor Gaikwad P, Banerjee R (2018). Advances in point-of-care diagnostic devices in cancers. Analyst.

[19] Crivianu-Gaita V, Thompson M (2016). Aptamers, antibody scFv, and antibody Fab’ fragments: An overview and comparison of three of the most versatile biosensor biorecognition elements. Biosens. Bioelectron..

[20] Becerril HA (2008). Evaluation of solution-processed reduced graphene oxide films as transparent conductors. ACS Nano.

[21] Suvarnaphaet P, Pechprasarn S (2017). Graphene-based materials for biosensors: A review. Sensors (Switzerland).

[22] Makaraviciute A, Ramanaviciene A (2013). Site-directed antibody immobilization techniques for immunosensors. Biosens. Bioelectron..

[23] Shen M, Rusling JF, Dixit CK (2017). Site-selective orientated immobilization of antibodies and conjugates for immunodiagnostics development. Methods.

[24] Zdarta J, Meyer AS, Jesionowski T, Pinelo M (2018). A general overview of support materials for enzyme immobilization: Characteristics, properties, practical utility. Catalysts.

[25] Luckarift HR, Spain JC, Naik RR, Stone MO (2004). Enzyme immobilization in a biomimetic silica support. Nat. Biotechnol..

[26] Zhang H, Jiang Z, Xia Q, Zhou D (2021). Progress and perspective of enzyme immobilization on zeolite crystal materials. Biochem. Eng. J..

[27] Joshi S, Guruprasad G, Kulkarni S, Ghosh R (2021). Reduced graphene oxide based electronic sensors for rapid and label-free detection of CEA and CYFRA 21-1. IEEE Sens. J..

[28] Ahuja, T., Mir, I. A., Kumar, D. & Rajesh. Biomolecular immobilization on conducting polymers for biosensing applications. *Biomaterials* **28**, 791–805 (2007). 10.1016/j.biomaterials.2006.09.046 17055573

[29] Tang H (2020). Nanopore-based strategy for selective detection of single carcinoembryonic antigen (CEA) molecules. Anal. Chem..

[30] Singh R, Hong S, Jang J (2017). Label-free detection of influenza viruses using a reduced graphene oxide-based electrochemical immunosensor integrated with a microfluidic platform. Sci. Rep..

[31] Miomandre F, Audebert P (2020). 1,2,4,5-Tetrazines: An intriguing heterocycles family with outstanding characteristics in the field of luminescence and electrochemistry. J. Photochem. Photobiol. C Photochem. Rev..

[32] Clavier G, Audebert P (2010). S-Tetrazines as building blocks for new functional molecules and molecular materials. Chem. Rev..

[33] Ghosh R, Midya A, Santra S, Ray SK, Guha PK (2013). Chemically reduced graphene oxide for ammonia detection at room temperature. ACS Appl. Mater. Interfaces.

[34] Chen W, Wang D, Dai C, Hamelberg D, Wang B (2012). Clicking 1,2,4,5-tetrazine and cyclooctynes with tunable reaction rates. Chem. Commun..

[35] Ruid M, Miguel AA, Cruz-quesada G, Rivera-utrilla J, Manuel S (2020). Hydrothermal Synthesis of rGO-TiO2 Composites as High-Performance UV Photocatalysts for Ethylparaben Degradation. Catalysts.

[36] Cruz M (2017). Bare TiO_2_ and graphene oxide TiO_2_ photocatalysts on the degradation of selected pesticides and influence of the water matrix. Appl. Surf. Sci..

[37] Du FP (2018). PEDOT:PSS/graphene quantum dots films with enhanced thermoelectric properties via strong interfacial interaction and phase separation. Sci. Rep..

[38] Jozghorbani M, Fathi M, Kazemi SH, Alinejadian N (2021). Determination of carcinoembryonic antigen as a tumor marker using a novel graphene-based label-free electrochemical immunosensor. Anal. Biochem..

[39] Zhang DS, Chang Z, Lv YB, Hu TL, Bu XH (2012). Construction and adsorption properties of microporous tetrazine-based organic frameworks. RSC Adv..

[40] Mattson EC (2014). Vibrational excitations and low-energy electronic structure of epoxide-decorated graphene. J. Phys. Chem. Lett..

[41] Abid SP, Islam SS, Mishra P, Ahmad S (2018). Reduced graphene oxide (rGO) based wideband optical sensor and the role of temperature, defect states and quantum efficiency. Sci. Rep..

[42] Honarmand M, Farhad-Mollashahi L, Nakhaee A, Nehi M (2016). Salivary levels of ErbB2 and CEA in oral squamous cell carcinoma patients. Asian Pac. J. Cancer Prev..

[43] Kulkarni S, Ghosh R (2021). A simple approach for sensing and accurate prediction of multiple organic vapors by sensors based on CuO nanowires. Sens. Actuators B Chem..

[44] Jennewein MF, Alter G (2017). The immunoregulatory roles of antibody glycosylation. Trends Immunol..

[45] Akbar R (2021). A compact vocabulary of paratope–epitope interactions enables predictability of antibody–antigen binding. Cell Rep..

