# Supplementary material for: An electronic biosensor based on semiconducting tetrazine polymer immobilizing matrix coated on rGO for carcinoembryonic antigen
Source: Sci Rep. 2022 Feb 22;12:3006. doi: 10.1038/s41598-022-06976-0 (PMC8863780; doi:10.1038/s41598-022-06976-0)
Supplement: Supplementary file 1 — Supplementary Figures. [file 41598_2022_6976_MOESM1_ESM.docx]

**Supplementary Information**

**A Novel Electronic Biosensor based on Semiconducting Tetrazine polymer Immobilizing Matrix coated on rGO for Carcinoembryonic Antigen**

Sowmya Joshi^1Ⴕ^, Aswani Raj K^2Ⴕ^, Rajeswara Rao M^2*^, Ruma Ghosh^1*^

^1^Department of Electrical Engineering, Indian Institute of Technology Dharwad, Karnataka – 580011, India

^2^Department of Chemistry, Indian Institute of Technology Dharwad, Karnataka – 580011, India

^Ⴕ^Both the authors have contributed equally

^*^Email: [rumaghosh@iitdh.ac.in](mailto:rumaghosh@iitdh.ac.in), [rajesh@iitdh.ac.in](mailto:rajesh@iitdh.ac.in)

**Fig. S1.** ^13^C NMR of **PhPTz** under different oxidation conditions.


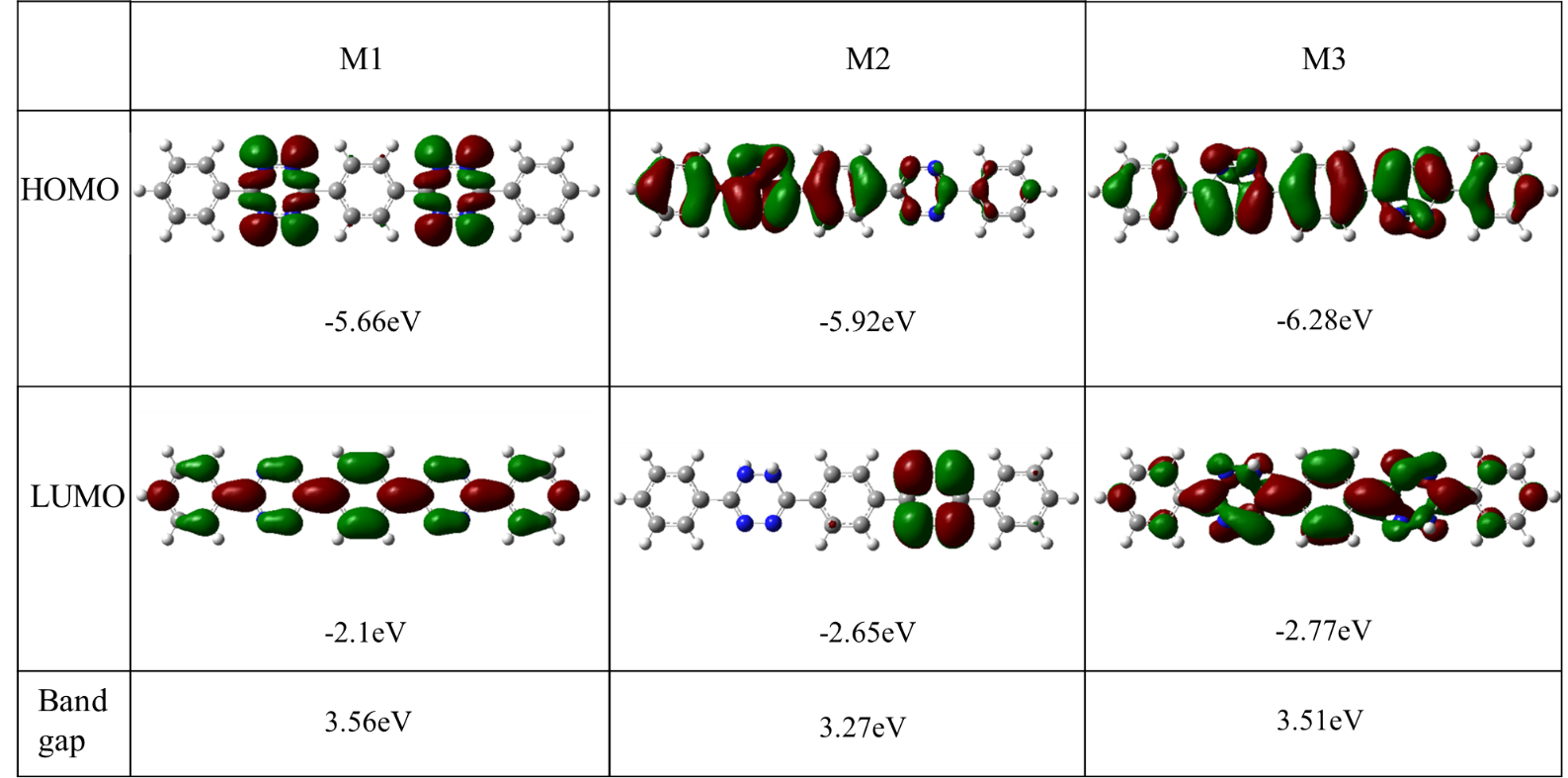


**Fig. S2.** DFT-optimized frontier molecular orbitals of Model compounds **M1**, **M2** and **M3.**

(b)

**Fig. S3 (a)** FTIR spectra of GO, rGO, MTz and PhUTz **(b)** I-V characteristics of GO and rGO samples**.**

**Fig. S4.** ^13^C NMR spectrum of unoxidized polymer **PhUTz.**

**Fig. S5.** Thermogravimetric analysis (TGA) curve of polymer **PhPTz** under heating rate of 10 ^o^C min^-1^.

**Fig. S6.** Kubelka munk plot of polymer **PhPTz.**

**Fig. S7.** Kubelka munk plot of GO**.**

**Fig. S8.** Kubelka munk plot of rGO**.**


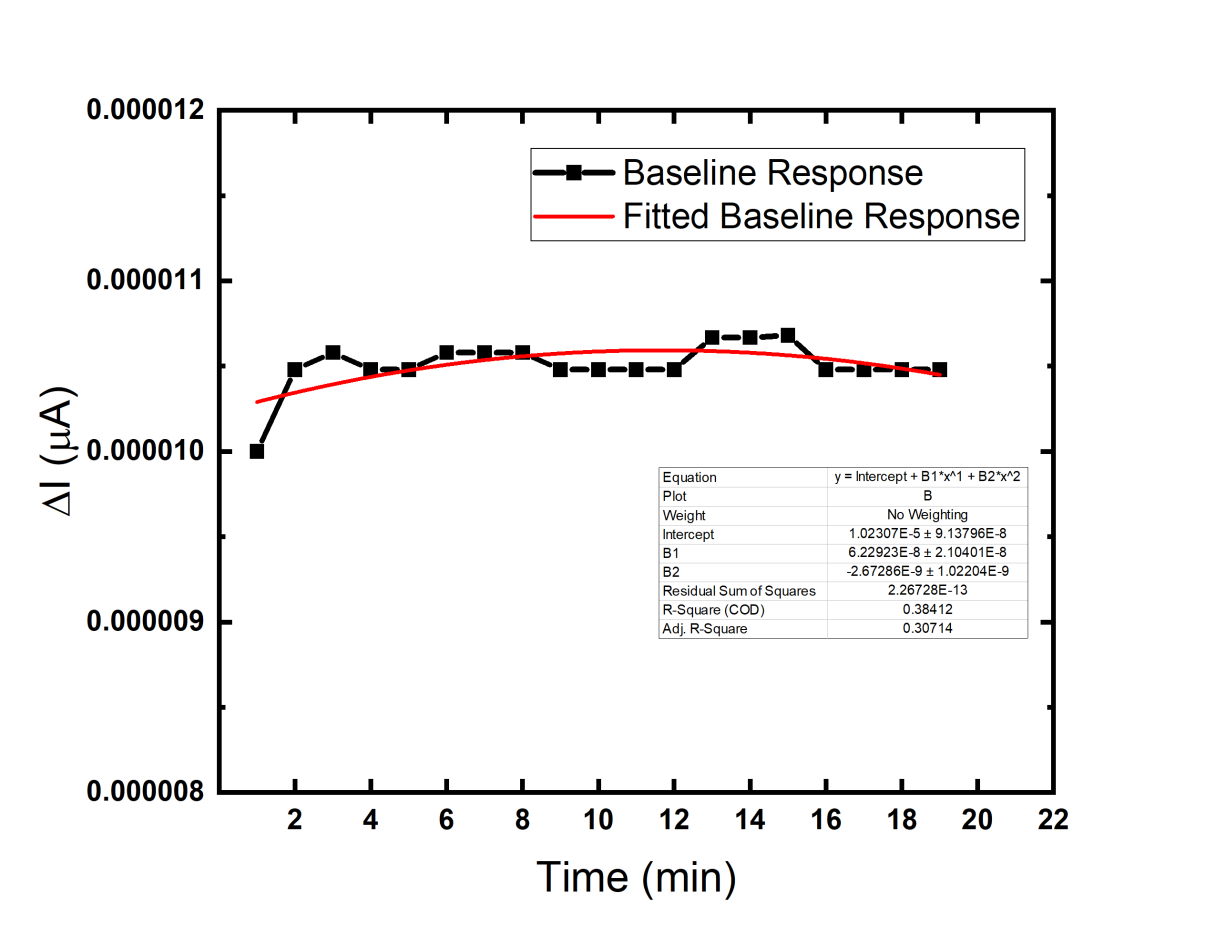

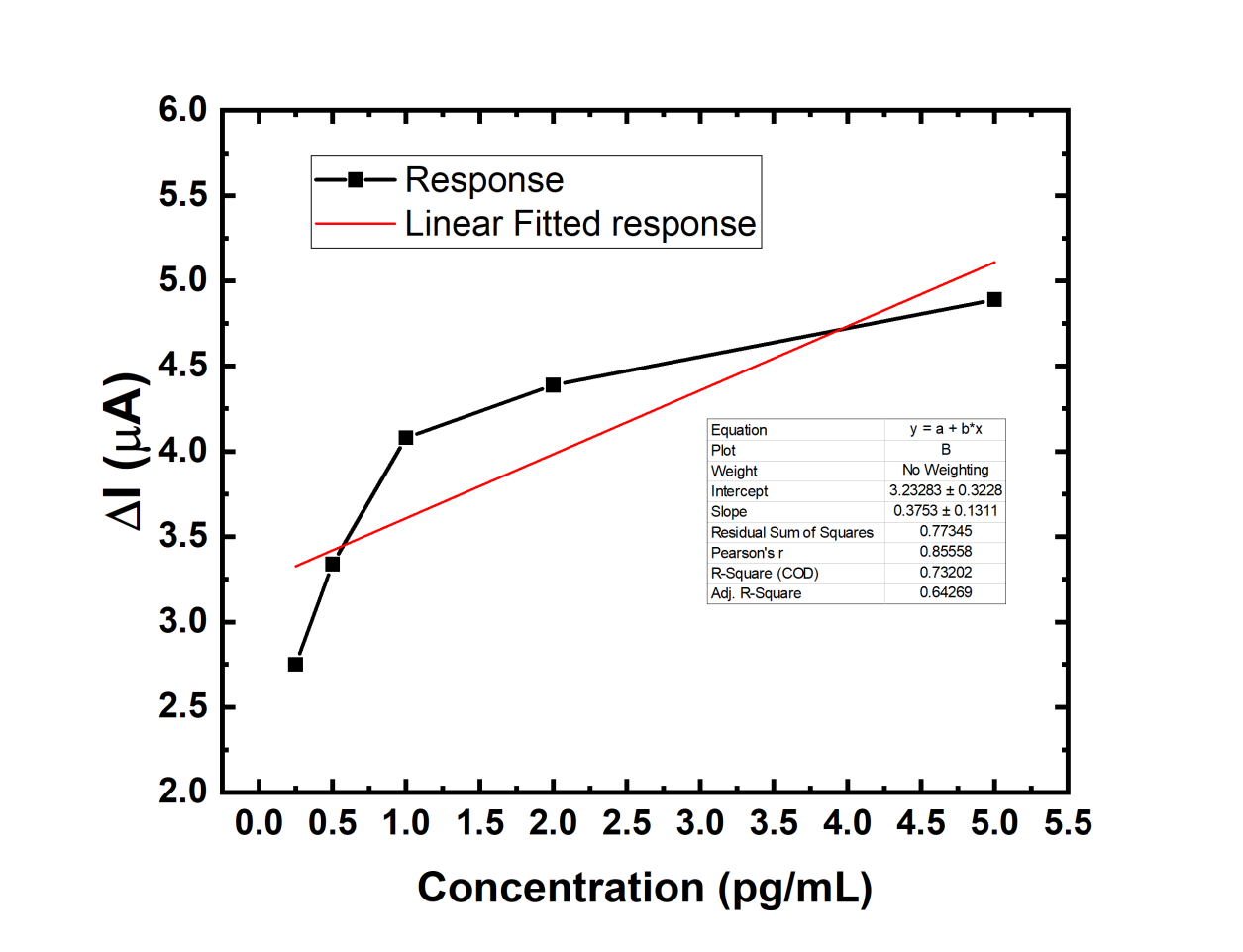


(a)

(b)

**Fig. S9.**  **(a)** Response and linear fitted response of the sensor to the blank measurements (baseline current) **(b)** Linear-fitted response of the sensors

In order to calculate the LOD, it was necessary to ascertain the slope from a linear fitted response Vs concentrations graph. The fitting was observed to be non-linear if we considered the whole range of the concentrations of CEA for which the sensors were tested. Hence, the linear fitting of the response Vs concentrations was done considering **only 0.25 to 5 pg/mL of CEA.**

**Fig. S10.** Response of the sensor fabricated by varying the volume of **PhPTz** in the device to 1 pg/mL of CEA at room temperature (25 °C).

**Fig. S11** Response of the sensors fabricated using **PhPTz** and **PhUTz** as antibody immobilizing agents to 20 ng/mL of CEA at room temperature


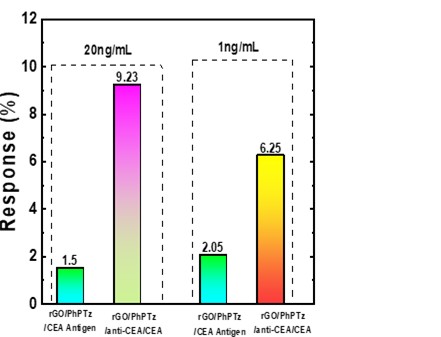


**Fig. S12** Response of the sensors fabricated of RGO and RGO/PhPTz layers to 20 ng/mL and 1ng/mL of CEA
